# Effect of Electroacupuncture on Short-Chain Fatty Acids in Peripheral Blood after Middle Cerebral Artery Occlusion/Reperfusion in Rats Based on Gas Chromatography–Mass Spectrometry

**DOI:** 10.1155/2022/3997947

**Published:** 2022-08-23

**Authors:** Xiaohua Ke, Qing Xiang, Pingli Jiang, Weilin Liu, Minguang Yang, Yihan Yang, Dan Shi, Lidian Chen, Jing Tao

**Affiliations:** ^1^College of Rehabilitation Medicine, Fujian University of Traditional Chinese Medicine, Fuzhou, Fujian 350122, China; ^2^Key Laboratory of Orthopedics & Traumatology of Traditional Chinese Medicine and Rehabilitation, Ministry of Education, Fuzhou, Fujian 350122, China; ^3^Fujian Collaborative Innovation Center for Rehabilitation Technology, Fuzhou, Fujian 350122, China; ^4^National-Local Joint Engineering Research Center of Rehabilitation Medicine Technology, Fujian University of Traditional Chinese Medicine, Fuzhou, Fujian 350122, China

## Abstract

Previous fundamental and clinical research has shown that electroacupuncture (EA) at the acupoints of Quchi (LI11) and Zusanli (ST36) can successfully alleviate motor dysfunction following stroke. Additionally, it has been discovered that gut microbiota and their metabolites play an essential role in stroke. However, the relationship between the metabolites of gut microbiota and the efficacy of EA is still unclear. Therefore, the aim of this study was to evaluate the mechanism of EA at LI11 and ST36 in the treatment of motor dysfunction after middle cerebral artery occlusion/reperfusion (MCAO/R) in model rats by comparing the differences and correlation between different short-chain fatty acids (SCFAs) and the recovery of motor function. The results indicated that EA at LI11 and ST36 acupoints enhanced the neurological function, motor function, and infarct volume of MCAO/R rats. The levels of acetic acid, propionic acid, and total SCFAs were considerably lower in the MCAO/R group than in the sham group (*P* < 0.05). Acetic acid, propionic acid, and total SCFA concentrations were substantially higher in the MCAO/R + EA group than in the MCAO/R group (*P* < 0.05). Finally, Pearson correlation analysis revealed that the propionic acid concentration was substantially favorably connected with the duration on the rotarod (*r* = 0.633 and *P* < 0.05) and highly negatively correlated with the modified neurological severity score (mNSS) (*r* = −0.698 and *P* < 0.05) and the percentage of cerebral infarct volume (*r* = −0.729 and *P* < 0.05). Taken together, these findings indicate that the increase in propionic acid may be one of the mechanisms and targets of EA at LI11 and ST36 acupoints to improve poststroke motor dysfunction in MCAO/R rats.

## 1. Introduction

Stroke is the second most common cause of human death [1]. Approximately 80% of survivors suffer from dysfunction, such as sensorimotor and cognitive impairments [2, 3], which seriously affects the patients' quality of life even after neurological rehabilitation [4]. Acupuncture is a traditional Chinese medical therapy with a long history and is widely used after ischemic stroke [5, 6]. Acupuncture is accomplished by inserting the tips of thin, stainless-steel needles on specific points (called acupoints) through the skin. Conventional acupuncture, also called “manual acupuncture,” involves the manipulation of the inserted needles by hand, such as lifting, thrusting, twisting, twirling, or other complex combinations. Electroacupuncture (EA) is a modification of this technique that stimulates acupoints with electrical current instead of manual manipulation and appears to have more consistently reproducible results in both clinical and research settings [7, 8]. Zusanli (ST36) and Quchi (LI11) are the acupoints most commonly used to clinically treat stroke in China. Our previous research showed that EA at ST36 and LI11 acupoints could relieve the inflammatory damage after stroke, reduce the volume of cerebral infarction, and promote neurological recovery through multiple channels and links [9–11]. However, its specific mechanism has not been fully explained thus far. A growing number of animal and clinical studies suggest that stroke risk and prognosis are significantly influenced by the gut microbiota composition [12–15]. Gut barrier function plays an important role between the human body and the gut microbiota, a fundamental premise for their symbiotic relationship [16]. In rodent studies, disruption of the intestinal barrier and intestinal dysfunction were found to occur after stroke [17–20]. Additionally, dysbiosis of gut microbiota after stroke affects mortality and functional recovery by activating T lymphocytes, which activate inflammatory immune responses [12, 21]. EA treatment has been shown to modulate gut microbiota and have significant positive effects on gut tissue [22, 23]. EA at ST36 acupoint can improve intestinal barrier function, protect intestinal barrier integrity, and reduce local intestinal inflammation [24–26]. Gut microbiota can interact with receptors in the intestinal wall and enteric nervous system through their metabolites, which then communicate with the brain via the vagal nervous system [27, 28]. Their metabolites can also diffuse into the blood circulation and play an endocrine role, sending signals to distant organs such as the brain [29–31]. Short-chain fatty acids (SCFAs) are organic fatty acids with fewer than six carbon atoms produced by bacteria through the fermentation of carbohydrates, including dietary and endogenous carbohydrates, and proteins. Among the SCFAs, acetic acid, propionic acid, and butyric acid account for the most significant proportion, and the ratio of the three is 3 : 1 : 1 [32–34]. Studies have demonstrated that SCFAs are key signaling molecules in the communication between the intestine and the brain [35] and have the ability to control neurodevelopment and blood–brain barrier integrity through neurotransmitters [36] and microglia [37] to regulate brain biochemistry and behavior [38, 39].

According to the latest research findings, central nervous system diseases, such as autism spectrum disorder, mood disorders, Alzheimer's disease, multiple sclerosis, Parkinson's disease, ischemic stroke, and poststroke cognitive impairment, can be affected by SCFAs [40–42]. In particular, acetic and propionic acids in SCFAs have been shown to readily cross the blood–brain barrier [43, 44] and affect brain function in development, health, and disease. Similarly, elevated circulating concentrations of SCFAs induce favorable therapeutic effects in the recovery period after chronic stroke. For example, SCFA treatment improved the course of experimental autoimmune encephalitis by promoting anti-inflammatory mechanisms and reducing axonal damage [45]. In a study by Sadler et al. [46], it was found that supplementation of middle cerebral artery occlusion/reperfusion (MCAO/R) mice with SCFAs significantly improved the motor function of the affected limb and that SCFAs induced connectivity changes in the contralateral cortex. Similarly, Park et al. [47] also found that SCFA treatment significantly reduced cerebral infarct volume, attenuated the inflammatory response, and improved sensorimotor deficits in female rats.

Therefore, in this study, we proposed utilizing EA at LI11 and ST36 acupoints to intervene in MCAO/R rats and measuring the changes in SCFAs in peripheral blood using GC–MS. The study aimed to examine the relationship between SCFAs and motor function after stroke, as well as to elaborate on the possible mechanism by which EA at LI11 and ST36 acupoints alleviates motor dysfunction after stroke.

## 2. Materials and Methods

### 2.1. Grouping of Rats

Healthy adult male Sprague–Dawley rats with a body weight of 300 ± 20 g were provided by Shanghai Laboratory Animal Co., Ltd. (Shanghai, China) (NO. SYXK 2020-0002). The animals were kept in a silent place under controlled environmental conditions: 12 h light/12 h darkness each day (light time: 7 : 00-19 : 00), 21-25 °C temperature, 40%-60% humidity, and noise level within 60 dB. Animals were made to adapt to the standardized laboratory facilities for one week. All rats had free access to water (autoclaved processing) and food (^60^Co irradiation sterilized breeding feed. NO. 2019060676. Beijing Huafukang Biotechnology Co., Ltd.) during the experiment, while the bedding (^60^Co irradiated corn cob bedding. Beijing Huafukang Biotechnology Co., Ltd.) and cages (NO. IsoRat900N.Tecniplast Group) were changed every other day. The animal experimental procedures were approved by the Animal Ethics Committee of Fujian University of Traditional Chinese Medicine. All experimental procedures were conducted according to the guidelines for the care and use of laboratory animals in biomedical research [48].

Applying a random number table method, the rats were randomly allocated into three groups (*n* = 6/group): (1) the sham group, in which the rats underwent identical surgical procedures and exposure to the internal carotid arteries, but an intraluminal occlusive monofilament was not inserted; (2) the MCAO/R group, in which the left internal carotid arteries were inserted with an intraluminal monofilament t; and (3) the MCAO/R + EA group, in which the surgical procedure was the same as that in the MCAO/R group and they received EA treatment.

### 2.2. MCAO/R Model Establishment

The MCAO/R model was established according to the Longa method [49]. Following 12 hours of preoperative fasting and free access to water during the period, the rats were anesthetized with 3% isoflurane in 67% N_2_ and 30% O_2_ and immobilized in the supine position. The skin was prepared in the middle of the neck of the rats and disinfected with alcohol iodine, and a longitudinal neck incision was made (median cervical longitudinal incision along the median line). The inner and outer muscles of the sternocleidomastoid muscle were separated to expose and bluntly isolate the left common, external, and internal carotid arteries. Subsequently, the superior thyroid and occipital arteries were separated and cauterized using preheated electrocautery to prevent bleeding. The pterygopalatine artery, a branch of the internal carotid artery, was ligated, and a noose was made at the distal end of the external carotid artery. Then, the common carotid artery and the internal carotid artery were clamped. An incision was cut between the two knots with microscissors, the blood vessel was cut off at the distal end of the external carotid artery, the external carotid artery was gently pulled to make it in the same line with the internal carotid, and the marked monofilament (Bosterembolus, Boster Biological Technology Co., Ltd., Wuhan, China) was slowly pushed along the direction of the external carotid artery through the incision until the resistance decreased (approximately 18 mm). Finally, the proximal end of the external carotid artery was ligated to fix the monofilament, and the arterial clips of the common carotid artery and the internal carotid artery were removed (Supplementary Figure S1 and Figure S2). After 90 minutes of ischemia, the monofilament was slowly pulled out, and the incision was sutured and disinfected with iodine after the operation. In the sham group, only the left common, external, and internal carotid arteries were separated, and no monofilament was inserted. During the surgery, the body temperature of the rats was maintained at 37 ± 1°C by an electric blanket until they recovered from anesthesia.

### 2.3. EA Treatment

In the MCAO/R + EA group, the acupoints of the rats were identified by a map developed by Hua et al. [50]. Briefly, two stainless acupuncture needles (diameter 0.3 mm) (Huatuo acupuncture needle, Suzhou Medical Appliance Factory, Suzhou, China) were perpendicularly inserted into LI11 (located in the depression lateral to the anterior aspect of the radius joint of the forelimb) and ST36 (located 5 mm below the knee joint of the hind-limb and 2 mm lateral to the anterior tubercle of the tibia) acupoints on the right paralyzed limb for a depth of 2-3 mm. Then, the parameters of the EA stimulator instrument (Model G6805; Shanghai Marine Instrument General Factory, Shanghai, China) were as follows: The peak voltage was 6 V, and the dilatational wave was 1/20 Hz, 30 minutes every time, once a day for 14 continuous days. EA treatment was administered between 9 : 00 am and 10 : 00 am daily. The sham group and the MCAO/R group were handled under the same settings at the same time every day and then returned to their cages without any treatment.

### 2.4. Neurological Assessment

**Figure 1:**
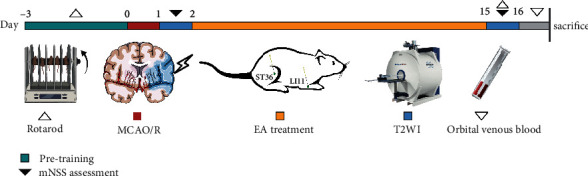
Testing timeline. Abbreviations: EA: electroacupuncture; MCAO/R: middle cerebral artery occlusion-reperfusion; mNSS: modified neurological severity score; and T2WI: T2-weighted imaging.

The mNSS [51] was performed to test neurological function by two investigators who were blinded to the group assignment [52] at one day and 15 days after reperfusion. The detailed procedure is shown in Figure 1. mNSS is a composite of four aspects: motor, sensory, balance, and reflection. The mNSS assessment was graded on a scale of 0 to 18 (a normal score is 0; the highest deficit score is 18). The higher the severity score was, the more serious the injury. For more details, see Supplementary Table S1.

### 2.5. Behavioral Assessment

The rotarod test was carried out on day 15 after MCAO/R induction by two researchers blinded to the experimental groups (Figure 1). All rats were assessed on approximately the same day. The rats were subjected to pretraining for 3 days twice a day before MCAO/R. Rats were placed on a rotarod (model: rotarod 47700, UGO BASILE, Italy), accelerating from 4 to 40 rpm over 5 minutes, with three repeated trials at 10-minute intervals. The average of the three recorded times of the rats remaining on the rotarod indicated motor function based on previously reported methods [53].

### 2.6. MRI Acquisition

MRI data (T2-weighted images) were acquired using a 9.4 T MRI Bruker Avance workstation and a 35-mm quadrature volume coil (Pharma scan, micro-MRI, Bruker Medizintechnik, Germany). Data scanning was performed for every group at 1 day and 15 days after reperfusion and analyzed in a blinded manner (Figure 1). To keep their physiological condition stable, the rats were anesthetized with isoflurane/O_2_ (3% for induction and 2% for maintenance). The rats were placed flat on a rodent bed with a warm water mat on top. Their vital signs (body temperature, respiratory, and heart rate) were monitored by a physiological detector in real time (SurgiVet V3395TPR, Smiths Medical Inc., St. Paul, MN).

T2-weighted imaging was captured using relaxation enhancement (RARE) sequences with the following parameters: repetition time (TR) = 2,500 ms, echo time (TE) = 33 ms, field of view (FOV) = 32 × 32 mm, average = 4, slices = 21, matrix = 256 × 256, slice thickness = 1 mm, bandwidth = 326087 Hz, echo spacing (ESP) = 10,000 ms, refocusing angle = 180°, excitation angle = 90°, echo train length (ETL) = 8, and k − zero = 3. ImageJ software (https://imagej.nih.gov/ij/) [54] was used to determine the cerebral infarction volume by using T2-weighted images. Infarct volume was calculated by the following formula [55]: infarct volume (%) = V_L_/V_W_ × 100%, where V_L_ (mm^3^) = scanned lesioned brain area (mm^2^) × scanned relative thickness (mm) and V_W_ (mm^3^) = scanned whole brain area (mm^2^) × scanned relative thickness (mm).

### 2.7. GC–MS Analysis

After 24 hours of the last EA treatment, rats in each group were anesthetized with isoflurane, and blood was collected from the orbital vein. One milliliter of blood was collected from each rat, 6 rats per group. The collected vein blood was allowed to stand at 4 °C for 45 minutes, centrifuged at 4 °C, and low speed (1300 g) for 10 minutes to remove blood cells and then centrifuged at 4 °C and high speed (13000 g) for 10 minutes to remove cell debris and precipitated particles from the sample solution. The upper serum was transferred with a pipette to a labeled EP tube and stored immediately at − 80 °C. Then, the sample was precisely transferred into 2-mL EP tubes, 50 *μ*L of 15% phosphoric acid was added accurately, and then, 10 *μ*L of 75 *μ*g/mL internal standard (isocaproic acid) solution and 140 *μ*L ether were homogenized for 1 minute, and centrifuged at 12000 rpm at 4 °C for 10 minutes. The supernatant was taken and tested on the machine.

The GC–MS system consists of a Vanquished UHPLC gas chromatograph coupled to a Q Exactive™ HF mass spectrometric detector (Thermo TRACE 1310-ISQ LT ThermoFisher, USA). The chromatographic conditions were as follows: Agilent HP-INNOWAX capillary column (30 mm × 0.25 mm, ID × 0.25 *μ*m); ion source temperature 230 °C; transmission line temperature 250 °C; and quadrupole temperature 150 °C. The column was started at 90 °C, increased to 120 °C at 10 °C/minute, increased to 150 °C at 5 °C/min, and finally increased to 250 °C at 25 °C/minute and held for 2 minutes. Helium was used as the carrier gas and passed through the column at a constant flow rate of 1 mL/minute. The injection volume was 1 *μ*L with a split ratio of 10 : 1. The injection port temperature was 250 °C.

Mass spectrometry conditions: the temperatures of the transmission line, injection port, and electron bombardment ionization (EI) source were set to 250 °C, 250 °C and 230 °C, respectively, with sim scan mode, and an electron energy of 70 eV.

### 2.8. Validation of Techniques

#### 2.8.1. Total Ion Flow Chromatogram

**Figure 2:**
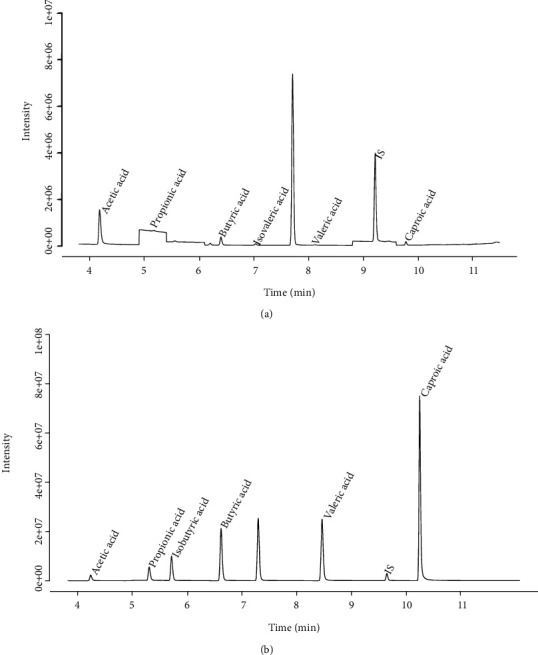
Total ion chromatogram of the SCFAs. (a) The total ion flow chromatogram of the SCFAs sample was obtained. (b) The total ion flow chromatogram was obtained from the prepared mixed SCFA standard sample.

As shown in Figures 2(a) and 2(b), the SCFAs were easily distinguished. The peak time of the internal standard (isocaproic acid) was 9.23 minutes, which was clearly separated from the other SCFA standard samples.

#### 2.8.2. Standard Curve and Limit of Quantification

After GC–MS detected the concentration series of the standards, the calibration curve was constructed with the concentration of the standards as the horizontal coordinate and the ratio of the peak area of the standards to the internal standard as the vertical coordinate. The linear regression equation of each substance obtained is shown in Supplementary Table S2 (correlation coefficient *r* > 0.99).

#### 2.8.3. Precision

Standard samples with a mixed standard concentration of 25 *μ*g/mL were injected eight times consecutively, and the intraday precision was calculated and expressed as the relative standard deviation (RSD). One 25 *μ*g/mL standard sample was processed each day for determination on the first, second, and third days and to calculate the interday precision, which was expressed as RSD. The results for the intraday and interday precision and repeatability are presented in Supplementary Table S2. The intraday precision ranged from 1.57% to 3.75%, and the interday precision ranged from 6.15% to 11.08%. The intraday and intraday precision and repeatability were all less than 15%, indicating that the method provided good precision.

#### 2.8.4. Recovery

Since each target substance is endogenous, the recovery rate = (actual value − theoretical value)/amount added × 100%. Supplementary Table S3 shows the recovery results of the SCFAs at different concentrations. The recoveries were in the range of 87.70% to 114.68%, suggesting that the method is accurate and meets the requirements for biological sample analysis.

#### 2.8.5. Quality Control (QC)

QC mainly consisted of stability evaluation and overlap chromatography. As shown in Supplementary Figure S3, the curves overlap well. Due to the volatility of SCFAs, samples should be processed as feasible after preparation to ensure the accuracy of the assessment. The transitory stability of the samples stored at 4 °C for 24 hours was examined in this work. For each batch of samples, the QC assay was performed at various pin intervals, and the method's stability was assessed by calculating the RSD of all QC samples using the peak area ratio of the standards to the internal standards. The results in Figure S3 show that the samples were stable enough to be analyzed further.

### 2.9. Statistical Analysis

The database was established by Excel 2019 for Windows (Microsoft Corporation, San Francisco, CA, USA), and all statistical tests were performed using IBM SPSS version 24.0 statistical software (SPSS Inc., Chicago, IL, USA). Continuous variables with a normal distribution are presented as the mean ± standard deviation; means of continuous normally distributed variables were compared by independent samples Student's *t* test and ANOVA (if the results of ANOVA were significant, least significant difference tests were used to compare the differences between the two means). Continuous variables that did not conform to a normal distribution are represented by the median and interquartile range. Nonparametric tests were used to compare the medians of non-normally distributed variables. *P* < 0.05 was regarded as statistically significant. SCFAs were identified using principal component analysis (PCA) and orthogonal projections with latent structures-discriminant analysis (OPLS-DA) by SIMCA-P14.0 software (https://umetrics.com/products/simca). In addition, 200 response displacement tests were performed to validate the model.

## 3. Results

### 3.1. EA Treatment Improved Neurological Function and Motor Function after MCAO/R

**Figure 3:**
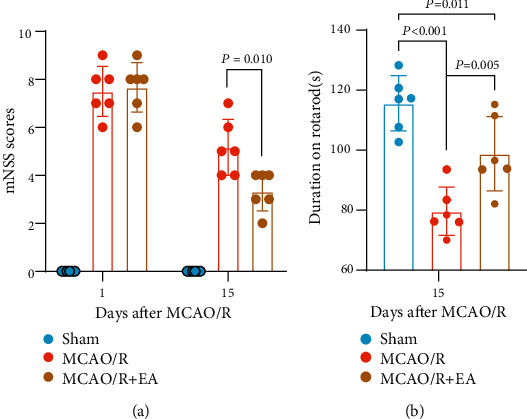
EA treatment positively affected neurological and motor function in MCAO/R rats. (a) The mNSS score of rats after 1 and 15 days of ischemia–reperfusion. The sham group had no neurologic deficits during the whole period (score of 0). (b) Performance of all rats at 15 days on the accelerating rotarod. Data are presented as the mean ± standard deviation, *n* = 6. Abbreviations: EA: electroacupuncture; MCAO/R: middle cerebral artery occlusion-reperfusion; mNSS: modified neurological severity score.

Neurological function was evaluated by mNSS scores.  Figure 3(a) shows that the mNSS score was dramatically increased in all MCAO/R rats compared with the control group. The mNSS scores of the MCAO/R + EA group and the MCAO/R group were not significantly different (*P* = 0.787 > 0.05) 1 day after ischemia–reperfusion, which indicated successful MCAO/R model establishment, whereas the mNSS scores in the MCAO/R + EA group were considerably reduced compared with those of the MCAO/R group (*P* = 0.010 < 0.05) at 15 days after MCAO/R.

All rats were subjected to rotarod tests. The duration on the rotarod decreased significantly in the MCAO/R group compared to the sham group but increased significantly in the MCAO/R + EA group compared to the MCAO/R group (*P* < 0.01, respectively; Figure 3(b)).

These results indicated that EA treatment could significantly improve the neurological and motor function of MCAO/R rats.

### 3.2. EA Treatment Reduced the Infarct Volume after MCAO/R

**Figure 4:**
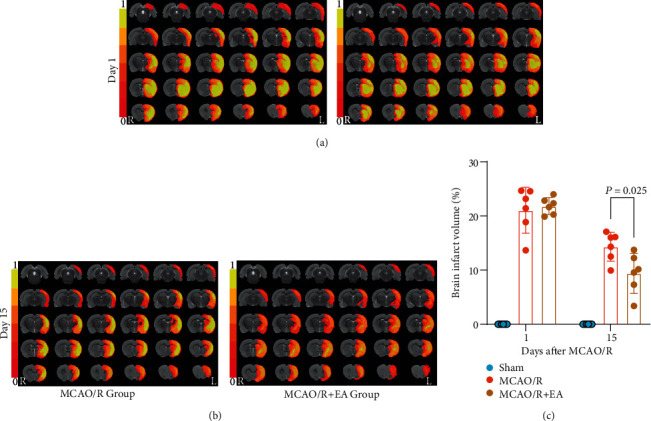
T2-weighted imaging signal changes before and after EA treatment. Changes in the infarct region and damaged volume were measured by T2-weighted imaging in the MCAO/R and MCAO/R + EA groups before (a) and after (b). Changes in the infarct region and damaged volume are represented as a percentage of the total brain volume. All data are presented as the mean ± standard deviation, *n* = 6. Abbreviations: EA: electroacupuncture; MCAO/R: middle cerebral artery occlusion-reperfusion; L: left; R: right.

T2-weighted imaging was used to assess the volume of the damage at 1 day and 15 days after reperfusion. The infarct lesions of the MCAO/R group and MCAO/R + EA group were overlaid on the rat standard template (Figures 4(a) and 4(b)). The color strips indicate the incidence of Paxinos and Watson spatial infarct lesions [56]. The higher the value is, the greater the probability of occurrence. Since there was no brain damage in the sham group, the incidence of infarction was 0%, so no color was shown on the standard template (Supplementary Figure S4). The volume of cerebral infarction was not significantly different between the MCAO/R and MCAO/R + EA groups at 1 day after reperfusion (*P* = 0.684 > 0.05), which indicated that the MCAO/R model was stable among the groups. We did not observe cerebral infarction in the sham group. As shown in Figure 4(c), the region of infarct lesions in the MCAO/R + EA group was improved at 15 days after reperfusion compared with the MCAO/R group. Furthermore, the percentage of the damaged volume was approximately 3.30% lower in the MCAO/R + EA group (9.40 ± 3.69% and *P* = 0.025) than in the MCAO/R group (12.70 ± 3.51%). These results suggest that EA treatment could reduce the lesion volume.

### 3.3. Multivariate Statistical Analysis

Metabolomic data are considered a multivariate dataset due to their high-throughput nature, where each substance in the sample represents a data dimension. Therefore, a series of multivariate pattern recognition analyses need to be performed on it, starting with PCA. PCA is used to form new characteristic variables by the linear combination of metabolite variables based on certain weights, categorize each group of data by prominent new variables (principal component), remove poorly reproducible samples (outlier samples), and remove abnormal samples (in the confidence interval-HotellingT2 ellipse outside the samples). The PCA score plot shows the degree of aggregation and dispersion of the samples. The closer the sample distribution points are, the more similar the composition and concentration of the variables/molecules in these samples are; conversely, the farther away the sample points are, the greater the variation.

**Figure 5:**
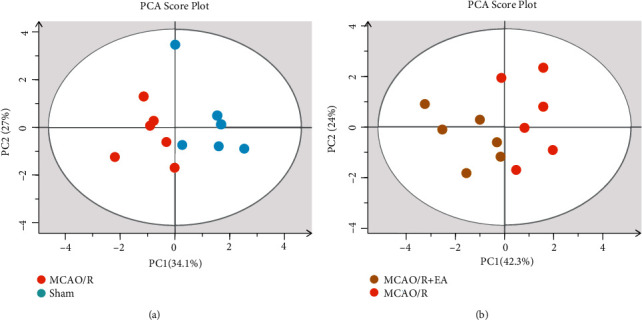
Principal component analysis score plots. (a) MCAO/R group vs. sham group, X interpretation rate = 0.611. (b) MCAO/R + EA group vs. MCAO/R group, X interpretation rate = 0.663. *R*^2^ (X) is the interpretation of the model. Usually, *R*^2^ higher than 0.5 is better. Abbreviations: EA: electroacupuncture; MCAO/R: middle cerebral artery occlusion-reperfusion; PCA: principal component analysis.

The concentrations of the seven SCFAs in the peripheral blood of rats in the sham, MCAO/R, and MCAO/R + EA groups was used as a variable in a multivariate statistical analysis (Figures 5(a) and 5(b)). An initial analysis of the global distribution and natural aggregation of the samples in each group was performed using principal component analysis. The cumulative value of the explained variance of X in the PCA score plot was 0.611 in the MCAO/R group and the sham group, and the cumulative value of the explained variance of X in the PCA score plot was 0.663 in the MCAO/R + EA group and MCAO/R group. The results of the principal component analysis indicated that the SCFAs differed among the three groups in the unsupervised mode.

**Figure 6:**
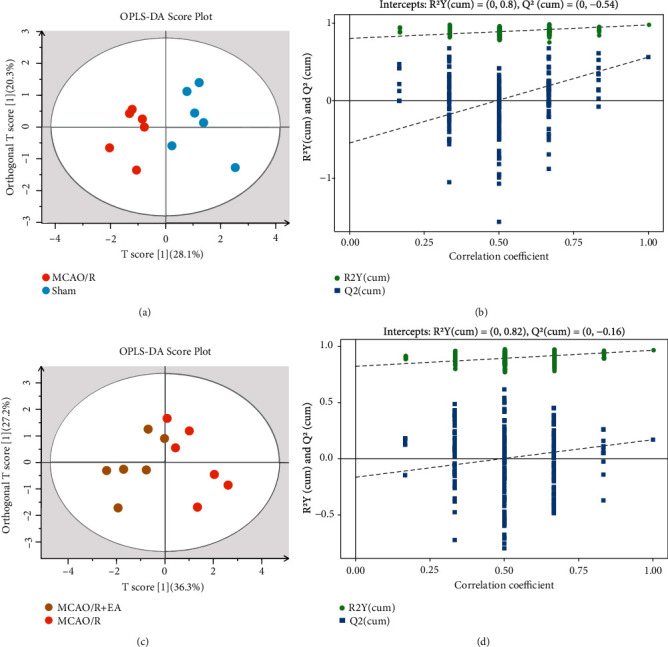
Score scatter plot and permutation test of the OPLS-DA model. (a), (c) OPLS-DA score plots of the MCAO/R group vs. the sham group (*X* interpretation rate = 0.485, *Y* interpretation rate = 0.978, and *Y* prediction rate = 0.564) and the MCAO/R + EA group vs. the MCAO/R group (*X* interpretation rate = 0.635, *Y* interpretation rate = 0.965, and *Y* prediction rate = 0.170). (b), (d) Permutation test plots of MCAO/R group vs. sham group (intercept of regression cure of *Y* interpretation rate = 0.80 and intercept of regression curve of *Y* prediction rate = −0.54) and MCAO/R + EA group vs. MCAO/R group (intercept of regression cure of *Y* interpretation rate = 0.82 and intercept of regression curve of *Y* prediction rate = −0.16). Abbreviations: EA: electroacupuncture; MCAO/R: middle cerebral artery occlusion-reperfusion; OPLS-DA: orthogonal projections to latent structures-discriminant analysis.

To better describe the discrepancy between the groups, a supervised OPLS-DA model was developed for analysis in this study. As seen in Figure 6(a), the cumulative value of the explained variance in the score plot was 0.485 for *X*, 0.978 for *Y*, and 0.564 for *Q*^2^ (cum) between the MCAO/R group and the sham group. As shown in Figure 6(c), the cumulative value of the explained variance in the PCA score plot was 0. 635 for *X*, 0.965 for *Y*, and 0.170 for *Q*^2^ (cum) between the MCAO/R + EA and MCAO/R groups.

According to the parameters validated by the procedure, the constructed mathematical model is stable and has a high predictive power. Furthermore, the *R*^2^ and *Q*^2^ values of the stochastic model were acquired by repeating the corresponding OPLS-DA model 200 times to verify whether the established model was overfitted and to assess the robustness of the model. In the comparison of the MCAO/R group and the sham group, the intercept of the regression curve of the *Y* interpretation rate was 0.80, and the intercept of the regression curve of the *Y* prediction rate was − 0.54; in the comparison of the MCAO/R + EA group and the MCAO/R group, the intercept of the regression curve of the *Y* interpretation rate was 0.82, and the intercept of the regression curve of the *Y* prediction rate was − 0.16 (Figures 6(b) and 6(d)). Above, as the replacement retention gradually decreases, the proportion of *Y* variables replaced decreases, and the random model of *Q*^2^ gradually decreases. This finding indicates that the original models of the MCAO/R group vs. the sham group and the MCAO/R group vs. the MCAO/R group have good robustness and no overfitting phenomenon.

Multivariate statistical analysis showed that the concentrations of SCFAs in peripheral blood differed among the sham group, MCAO/R group, and MCAO/R + EA group. Therefore, the OPLS-DA model developed in this study accurately differentiated the concentrations of SCFAs in peripheral blood among the three groups.

### 3.4. Effect of EA Treatment on the Peripheral Blood Concentrations of SCFAs afterMCAO/R

**Figure 7:**
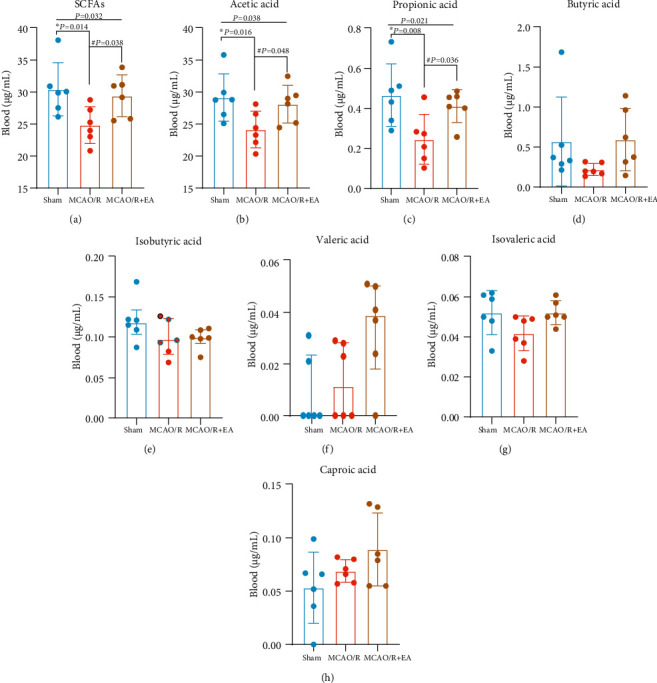
Concentrations of SCFAs in the peripheral blood of the sham group, MCAO/R group, and MCAO/R + EA group. The data (a–d, g, and h) are presented as the mean ± standard deviation, and the data (e and f) are presented as the median ± quartile, *n* = 6. One-way analysis of variance (a–d, g, and h) and the Kruskal–Wallis test (e and f) were used. Abbreviations: EA: electroacupuncture; MCAO/R: middle cerebral artery occlusion-reperfusion.

SCFAs are important energy sources and signaling molecules in the body, and they have important physiological roles. In this study, the established method was used to evaluate the level of SCFAs in the peripheral blood of rats in the sham group, MCAO/R group, and MCAO/R + EA group 14 days after EA treatment. As shown in Figure 7, the assay results indicated that the concentrations of acetic acid (*P* = 0.016), propionic acid (*P* = 0.008), and total SCFAs (*P* = 0.014) were much lower in the MCAO/R group than in the sham group (Figures 7(a)–7(c)). The concentrations of acetic acid (*P* = 0.048), propionic acid (*P* = 0.036), and total SCFAs (*P* = 0.038) were greatly increased in the MCAO/R + EA group compared with the MCAO/R group (Figures 7(a)–7(c)), and the concentrations of isobutyric acid, isovaleric acid, and valeric acid also tended to increase; however, no statistical significance was observed (Figures 7(e)–7(g)). Taken together, these findings showed that EA treatment may play a key role in stroke recovery by influencing SCFA concentration levels.

### 3.5. Correlation Analyses

**Figure 8:**
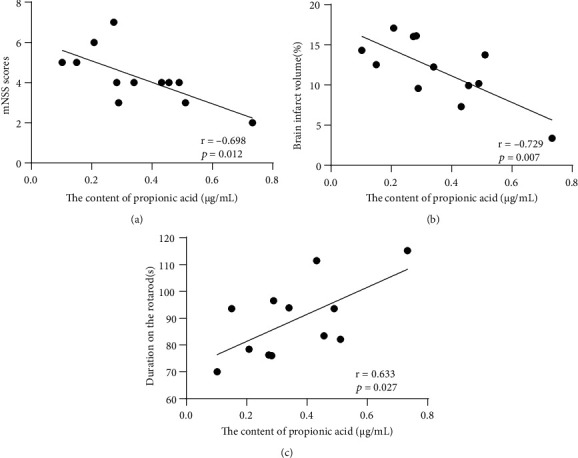
Correlation between the concentrations of propionic acid and clinical features. Pearson's correlation analyses between the concentrations of propionic acid and the value in (a) mNSS, (b) brain infarct volume, and (c) rotarod. Significant correlations were determined based on < –0.5 Pearson *r* < 0.5 and *P* < 0.05. Abbreviations: mNSS: modified neurological severity score.

To examine whether SCFAs were correlated with the clinical features of stroke and to determine whether the changes in SCFA concentration, and clinical features of stroke following EA treatment are associated, we further analyzed the relationships between SCFAs (total SCFAs, acetic acid and propionic acid) and time on rotarod, mNSS tests, and the percentage of cerebral infarct volume using Pearson correlation (Supplementary Table S4). The correlation analyses indicated that the concentration of propionic acid was strongly positively correlated with the duration of the rotarod (*r* = 0.633 and *P* < 0.05) (Figure 8(c)) and was strongly negatively correlated with the mNSS scores (*r* = −0.698 and *P* < 0.05) and the percentage of brain infarct volume (*r* = −0.729 and *P* < 0.01) (Figures 8(a) and 8(b)).

## 4. Discussion

Stroke is a severe health hazard and a significant cause of neurological dysfunction in adults. Approximately 80% of stroke patients experience permanent dysfunction, and motor dysfunction is the most significant symptom [57]. It seriously affects patients' quality of life and places a heavy burden on families and society. It has a significant impact on quality of life and places a significant burden on families and society. EA is widely used in clinical practice because it is simple, easy to perform, and has few side effects. EA has been shown to significantly improve the functional recovery of poststroke patients and animals [58–60]. Moreover, EA is also a complementary therapy for postoperative motor dysfunction, and EA at LI11 and ST36 acupoints can greatly enhance the motor function of stroke model rats [61]. This study showed that, compared to the MCAO/R group, the mNSS score and the rotarod duration of the rats in the MCAO/R + EA group were significantly better than those of the MCAO/R group after EA treatment, which indicated that EA improved their motor function. The results of this study are consistent with the previous research results of our team [9, 62].

Immediately after the treatment, we found no cerebral infarction in the sham group by T2-weighted imaging, while both the MCAO/R group and the MCAO/R + EA group had different degrees of cerebral infarction. After EA treatment, the percentage of brain damage volume in the MCAO/R + EA group was approximately 3.30% lower than that in the MCAO/R group. This further indicates that EA treatment can promote a reduction in cerebral infarct volume and improve the motor function of stroke rats. However, the mechanism of EA treatment for poststroke motor function is unknown, and SCFAs produced by gut microbiota are expected to genera te new ideas for mechanistic studies of EA.

SCFAs are metabolites of gut microbiota, and dysregulation of gut microbiota after stroke can affect the production of SCFAs. Studies have suggested that SCFAs have an essential role in preventing and treating central nervous system disorders [63, 64]. Tan et al. [65] studied the gut microbiota and fecal SCFA profile in Chinese acute ischemic stroke patients with different stroke severities, and they found a significant imbalance of gut microbiota with the presence of SCFA deficiency and a leaky gut. Moreover, studies have shown that cynomolgus monkey have dysbiosis and intestinal mucosal damage after cerebral infarction, and plasma SCFA levels were observed to decrease at 6 and 12 months after MCAO surgery, indicating chronic gut microbiotadysregulation may also hinder the production of SCFAs [66]. Similarly, we found that after EA treatment, rats recovered better motor function and had significantly higher total SCFA levels in peripheral blood than the MCAO/R group. Therefore, SCFAs may be a therapeutic target to improve the prognosis of stroke.

Acetic acid and propionic acid are the main components of SCFAs [66], which have been demonstrated to readily pass the blood–brain barrier and regulate brain function in terms of disease, development, and health [43, 44]. Acetic and propionic acids are not only sources of energy for the intestinal epithelium, but they are also essential to intestinal immunity [67]. A clinical study discovered that low acetic acid levels were especially noticeable in individuals with acute ischemic stroke and were substantially associated with an elevated likelihood of 90-day dysfunction [65]. Zhou et al. [68] intranasally administered a moderate dose (12.5 mg/kg) of acetic acid to mice by MCAO and found that it reduced the infarct volume and improved neurological function in MCAO mice. So et al. [69] showed that acetic acid could prevent further increases in infarct volume in the ischemic semidark zone of MCAO mice and improve their motor function by reducing the harmful chronic neuroinflammation mediated by microglia and macrophages. This study also showed that acetic acid increased during the recovery of motor function in stroke rats after EA treatment.

Few studies have examined the correlation between propionic acid and stroke prognosis. The present study found that propionic acid levels were positively correlated with the rotarod time and strongly negatively correlated with the mNSS score and the percentage of cerebral infarct volume. According to some studies, SCFAs can alter lymphocyte function and regulate the polarization of lymphocyte subsets, such as the ratio of anti-inflammatory T cells (Treg) to proinflammatory TH17 cells [47, 70] and their migratory behavior between organs [71], thereby indirectly affecting the inflammatory environment of the brain. Because propionic acid is one of the SCFAs that reaches considerable amounts in the systemic circulation under normal settings, our findings lend credence to the concept that propionic acid generated by the gut microbiota is a key anti-inflammatory mechanism. In addition, GPR43 binds to propionic acid, regulates a variety of inflammatory cellular responses, increases the expression of anti-inflammatory cytokines (IL-10), and reduces the expression of proinflammatory factors (IL-1*β*, IL-6, and IL-17) [72].

As a weak organic acid, when propionic acid accumulates passively in the cells of the central nervous system, there is a decrease in intracellular pH, which increases the synthesis and release of transmitters that affect motor activity [73–75]. The present study found an increased propionic acid level in the peripheral blood of stroke rats after EA treatment. An increase in propionic acid also promotes intracellular calcium release and elevates nitric oxide, altering neurotransmission in brain regions associated with motor behavior [74, 76]. Propionic acid was found to be closely associated with poststroke motor function in this study, and the underlying mechanism may be that EA treatment modulates gut microbial homeostasis, thereby increasing propionic acid levels in the peripheral blood; this, in turn, may accelerate the recovery of motor function after stroke by reducing the inflammatory response and enhancing the immune response.

In conclusion, the present study revealed a strong correlation between the improvement of motor function in rats after stroke and the production of SCFAs, particularly acetic acid and propionic acid, by the gut microbiota. Our findings might provide new insights into the mechanisms of poststroke motor dysfunction and lead to the identification of new targets for improving stroke recovery. EA treatment poststroke may improve motor function by increasing the levels of acetate and propionic acid or by stabilizing the gut microbiota producing these substances, which improves the recovery of motor function after stroke.

Of course, the specific pathways and molecular mechanisms related to the use of EA to increase propionic acid and acetic acid for motor function recovery in MCAO/R rats and whether propionic and acetic acids are prognostic markers need to be further investigated in our future studies. In addition, the first pathophysiological mechanism after stroke occurs in the brain tissue, not the gut. In future studies, we will investigate the specificity of acetic acid and propionic acid in the brain simultaneously to determine whether propionic acid passes through the blood–brain barrier to further clarify the link between propionic acid and motor function recovery after stroke. The current study also provides data to support subsequent clinical studies on whether EA treatment and SCFAs acting alone or together in MCAO/R rats can improve their motor function.

## Data Availability

The data are included in the article/supplementary material; further questions should be directed to the corresponding author.

## Supplementary Materials

Supplementary Materials Figure S1. Schematic diagram of the carotid artery and monofilament insertion in rats. Figure S2 (a) Exposure of common, internal and external carotid arteries; (b) ligation of the distal common and external carotid arteries and a live knot in the proximal external and internal carotid arteries; (c) inserting the monofilament; and (d) fixed the monofilament. Figure S3. Overlapping chromatograms of QC samples. Figure S4. T2-weighted imaging signal changes before and after sham group. Table S1. Modified neurological severity score (mNSS). Table S2. Regression equation, linear range, precision, repeatability, limit quantitation, and stability for the determination of the SCFAs in peripheral blood (*n* = 6). Table S3. Rate for the recovery for the determination of standard sample (*n* = 6). Table S4. Pearson correlation analysis total SCFAs, acetic acid and propionic acid and time on the rotarod, mNSS tests, and the percentage of cerebral infarct volume.
