# Supplementary material for: Effect of Electroacupuncture on Short-Chain Fatty Acids in Peripheral Blood after Middle Cerebral Artery Occlusion/Reperfusion in Rats Based on Gas Chromatography–Mass Spectrometry
Source: Mediators Inflamm. 2022 Aug 23;2022:3997947. doi: 10.1155/2022/3997947 (PMC9427317; doi:10.1155/2022/3997947)
Supplement: Supplementary Materials — Figure S1. Schematic diagram of the carotid artery and monofilament insertion in rats. Figure S2 (a) Exposure of common, internal and external carotid arteries; (b) ligation of the distal common and external carotid arteries and a live knot in the proximal external and internal carotid arteries; (c) inserting the monofilament; and (d) fixed the monofilament. Figure S3. Overlapping chromatograms of QC samples. Figure S4. T2-weighted imaging signal changes before and after sham group. Table S1. Modified neurological severity score (mNSS). Table S2. Regression equation, linear range, precision, repeatability, limit quantitation, and stability for the determination of the SCFAs in peripheral blood (n = 6). Table S3. Rate for the recovery for the determination of standard sample (n = 6). Table S4. Pearson correlation analysis total SCFAs, acetic acid and propionic acid and time on the rotarod, mNSS tests, and the percentage of cerebral infarct volume. [file 3997947.f1.zip › Supplementary materials .docx]

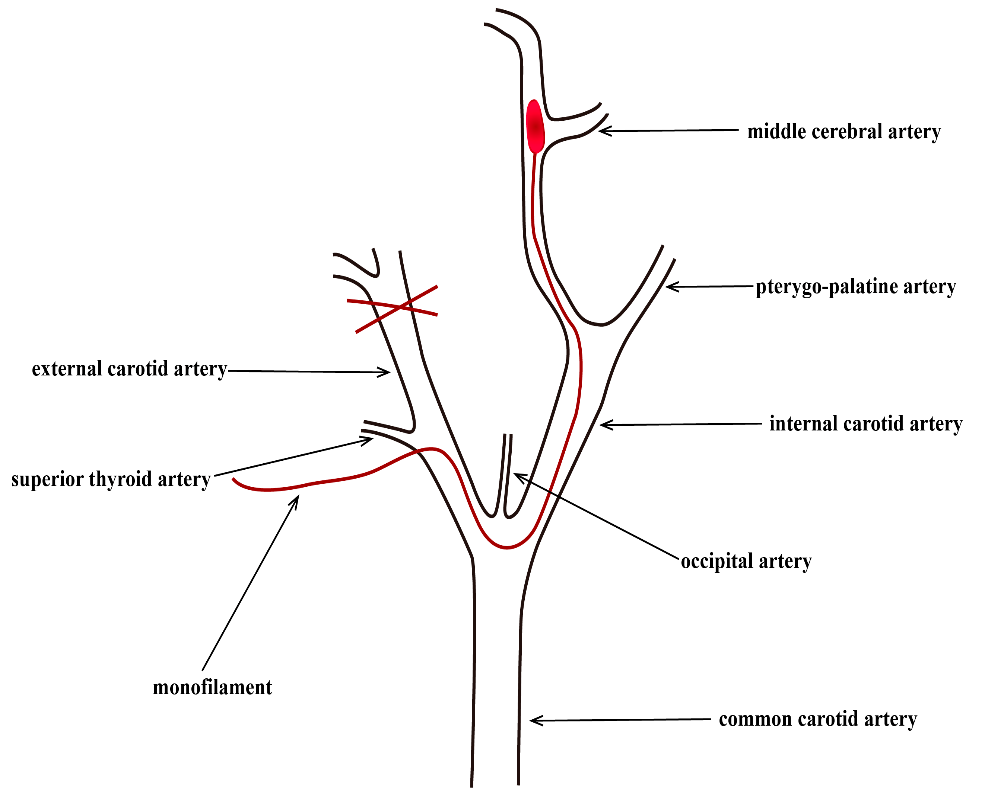


**Figure. S1|** Schematic diagram of the carotid artery and monofilament insertion in rats


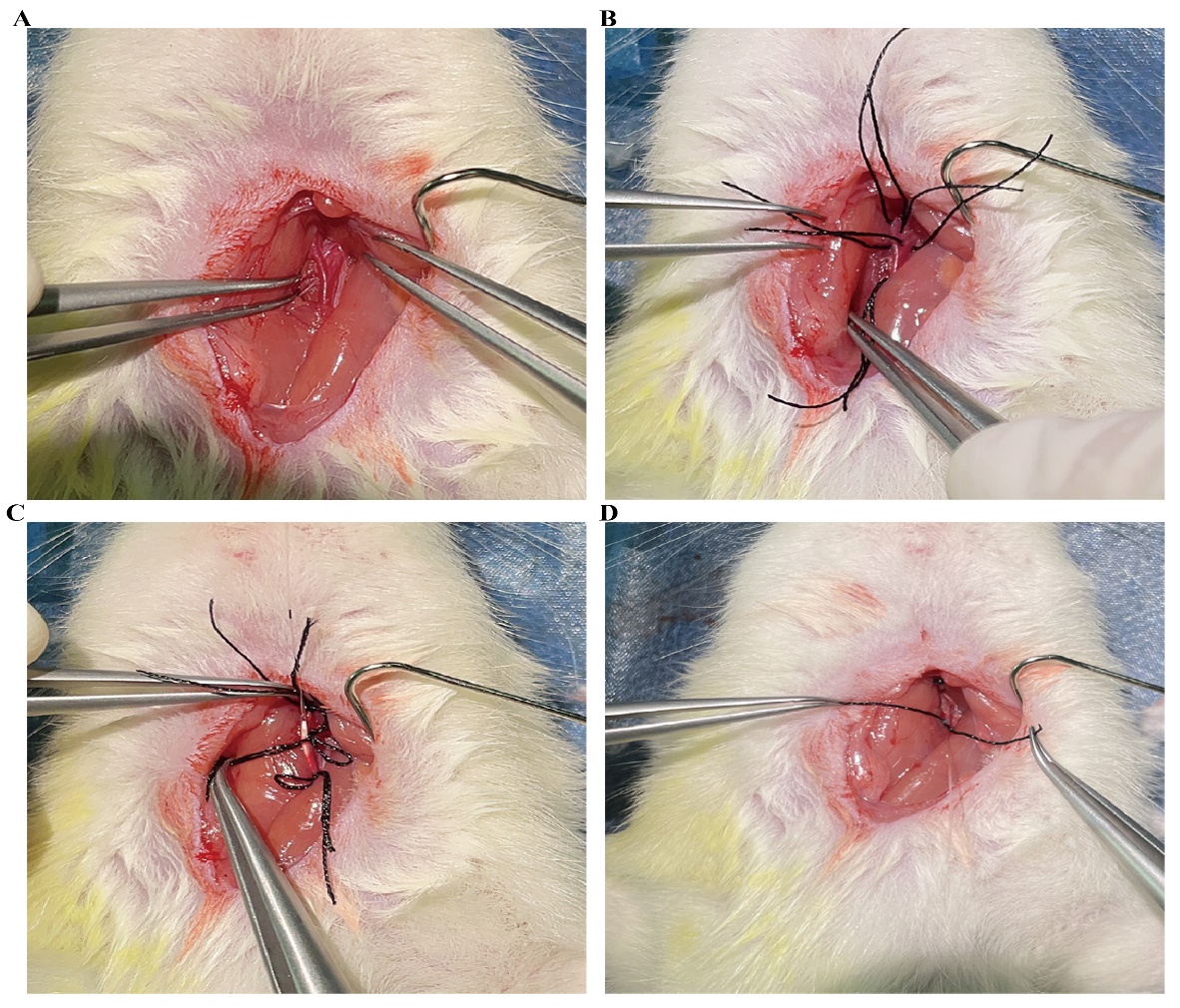


**Figure. S2|** (A)Exposure of common, internal and external carotid arteries;(B) Ligation of the distal common and external carotid arteries and a live knot in the proximal external and internal carotid arteries;(C) Inserting the monofilament;(D) Fixed the monofilament.


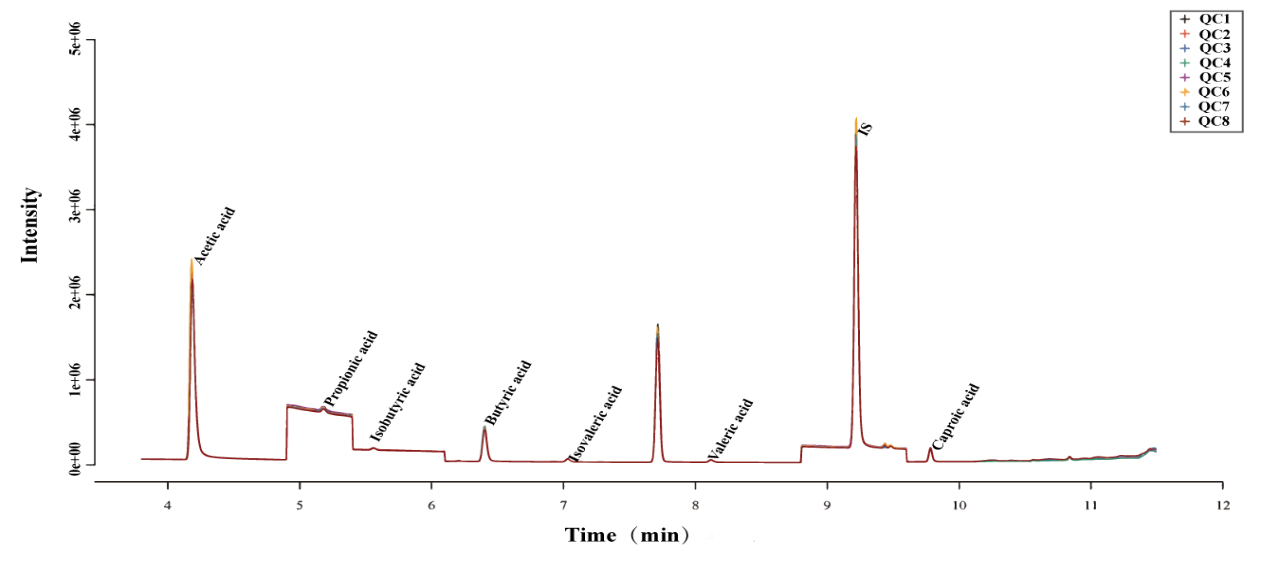


**Figure. S3|** Overlapping chromatograms of QC samples.


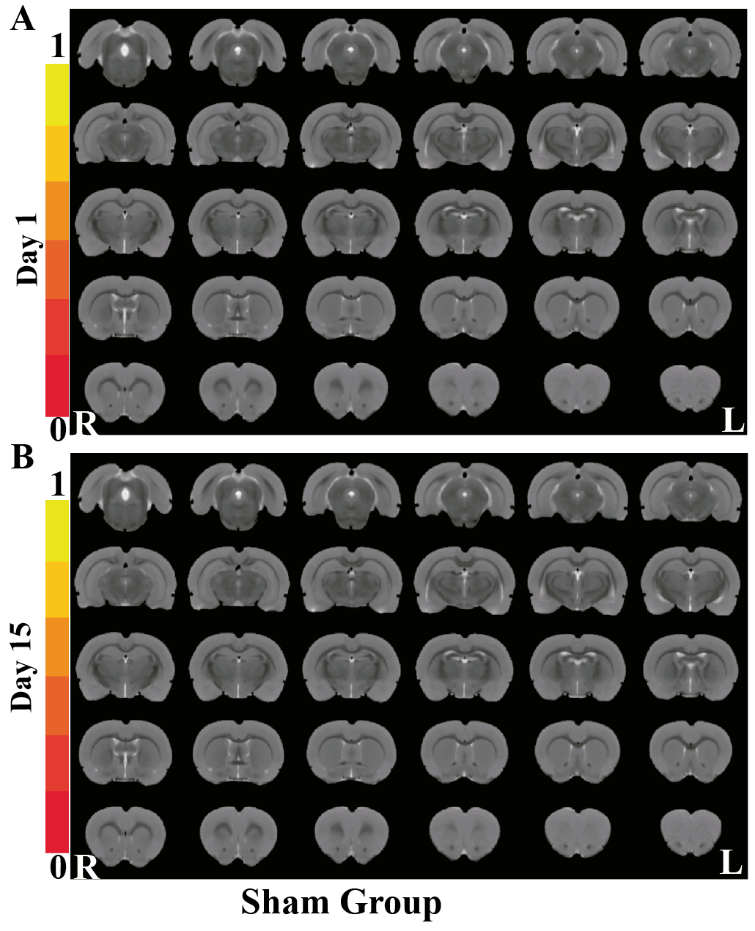


**Figure. S4|** T2-weighted imaging signal changes before and after sham group.

**Table S1 Modified neurological severity score (mNSS)**

| Tests | Points |
| --- | --- |
| **Motor tests** |  |
| Raising by the tail  1 forelimb flexion  1 hindlimb flexion  1 head moved >10° to vertical axis within 30 s | 3 |
| Placing on the floor 0 normal walk  1 inability to walk  2 circling  3 fall down | 3 |
| **Sensory tests** |  |
| 1Placing test (visual and tactile)  1Proprioceptive test  (deep sensation, pushing the paw against the table edge to stimulate limb muscles) | 2 |
| **Beam balance tests**  0 Balances with steady posture  1 Grasps side of beam  2 Hugs the beam and one limb falls down from the beam  3 Hugs the beam and two limbs fall down from the beam, or spins on beam (>60 s)  4 Attempts to balance on the beam but falls off (>40 s)  5 Attempts to balance on the beam but falls off (>20 s)  6 Falls off: No attempt to balance or hang on to the beam (<20 s) | 6 |
| **Reflexes/movements**  1 Pinna reflex (head shake when touching the auditory meatus)  1 Corneal reflex (eye blink when lightly touching the cornea with cotton)  1 Startle reflex (motor response to a brief noise from snapping a clipboard paper)  1 Seizures, myoclonus, myodystony | 4 |
| Maximum score points | 18 |

The original neurological severity score by Chen et al[1]. was developed to evaluate the neuroprotective effect of mesenchymal stem cells after MCAO in rats. It is a composite of motor, sensory, reflex and balance tests resulting in a deficit score from 0 (normal function) to 18 (maximal deficit) according to the following scoring scheme

**1.Raising by the tail**

Rats were suspended by their tails for one metre and observed for deviation and flexion of the head and front and hind limbs. Normal rats with no angle or an angle ≤10° between the head and the vertical axis of the body for a short period of time and with the limbs extended towards the ground were scored as 0. If there was flexion of the front limbs, flexion of the hind limbs and deviation of the head from the vertical axis >10°within 30 seconds, the abnormality was scored as 1 point respectively.

**2.Walking test**

Rats were placed on a large soft mat and observed for free walking behaviour, with scores of 0- 3 for normal walking, inability to walk in a straight line, turning in circles towards the mildly paralysed side and tipping towards the mildly paralysed side.

**3.Sensory tests**

**Visual test:**The experimenter holds the animal in his hand, leaving its front paws hanging in the air, and slowly tilts it 45° closer to the table from 10 cm above the table (when the table is located in front of the rat or on the affected side), the rat's normal response is to grasp the table immediately with the front limbs (0 point), while the injured rat shows a delayed limb response (1 point), and a delayed limb response on any of the three trials of front, left or right side, i.e. or is considered as a visual experiment impairment (1 point).

**Tactile test:** the rat's head was lifted upwards at 45° and the front paws were suspended in the air, at this point the rat should neither be able to see nor touch the table with its whiskers, the dorsal side of the front paws was used to lightly touch the table, the stimulation depth only reached the skin and hair, the animal's response and score were the same as the visual experiment, tactile stimulation was also divided into frontal and lateral stimulation.

**Proprioceptive test:** The rat is placed on the table with its head facing the edge of the table and gently pushed towards the edge of the table from behind, normally the rat will grasp the edge of the table and the affected limb will fall off; or the rat is placed on the table with the affected limb near the edge of the table and when the rat is pushed towards the edge of the table, the placement of the front and hind limbs on that side is observed and the normal rat can grasp the edge of the table while the affected rat's front and hind limbs cannot grasp the edge of the table.

**4.Beam balance tests**

A 170 cm long, 2 cm wide square wooden bar, placed flat at 70 cm from the ground, the rat was placed on the balance beam to observe the balance and scored according to the scale in Table S1.

**5. Reflexes/movements**

**Pinna reflex:** place the rat on a table top and touch the external ear canal with the hand; normal rats have a head shake response (0 point) and no head shake response is considered abnormal (1 point) .

**Corneal reflex:** blinking when the cornea is lightly touched with a cotton wool (0 point), abnormal if no blinking response (1 point).

**Startle reflex:** when a rat is placed on a table and a cardboard that makes noise is flicked rapidly close to the ear, an escape movement response is seen in normal rats (0 point), absence of escape movement is considered abnormal (1 point).

**Seizures, myoclonus and myodystony:** if any one of the three symptoms is present (1 point), normal if none of the three are present (0 point).

**Reference**

1.Chen J, Li Y, Wang L, Zhang Z, Lu D, Lu M, et al. Therapeutic benefit of intravenous administration of bone marrow stromal cells after cerebral ischemia in rats. *Stroke*. 2001;32:1005-1011.

**Table S2** Regression equation, linear range，precision，Repeatability ,limit Quantitation and stability for the determination of the SCFAs in peripheral blood(n=6)

| SCFAs | Retention time(min) | Quantitative ion | Regression equation | Correlation coefficient(r) | Linear range(μg/mL) | Intra-day precision RSD(%) | Inter-day precision RSD(%) | Repeatability  RSD(%) | Limit Quantitation(μg/mL) | Stability RSD(%) |
| --- | --- | --- | --- | --- | --- | --- | --- | --- | --- | --- |
| Acetic acid | 4.19 | 60 | y=0.0195x+0.0234 | 0.9918 | 0.02~100 | 2.89 | 11.08 | 2.35 | 0.02 | 2.58 |
| Propionic acid | 5.19 | 74 | y=0.0388x+0.002 | 0.9967 | 0.02~100 | 2.37 | 7.41 | 1.84 | 0.02 | 7.69 |
| Butyric acid | 5.57 | 73 | y=0.0704x-7e-4 | 0.9960 | 0.02~100 | 2.16 | 6.15 | 3.50 | 0.02 | 14.72 |
| Isobutyric acid | 6.41 | 60 | y=0.1528x+0.0066 | 0.9983 | 0.02~100 | 2.03 | 8.28 | 7.19 | 0.02 | 0.75 |
| Valeric acid | 7.04 | 60 | y=0.1817x-2e-4 | 0.9988 | 0.02~100 | 1.72 | 9.51 | 6.86 | 0.02 | 3.82 |
| Isovaleric acid | 8.13 | 60 | y=0.1782x+0.0012 | 0.9972 | 0.02~100 | 1.57 | 8.99 | 11.80 | 0.02 | 2.31 |
| Caproic acid | 9.79 | 60 | y=0.4456x+0.0054 | 0.9947 | 0.02~100 | 3.75 | 6.57 | 7.31 | 0.02 | 0.75 |

**Table S3** Rate for the recovery for the determination of standard sample(n=6)

|  | Acetic acid | Propionic acid | Butyric acid | Isobutyric acid | | Valeric acid | Isovaleric acid | | Caproic acid |
| --- | --- | --- | --- | --- | --- | --- | --- | --- | --- |
| LQC(μg/mL) | 1 | 0.1 | 0.2 | 0.05 | | 0.2 | 0.05 | | 0.05 |
| The rate for the recovery | 92.02% | 97.35% | 104.81% | 101.81% | | 104.02% | 99.78% | | 109/59% |
| MQC(μg/mL) | 10 | 1 | 1 | 1 | | 1 | 1 | | 1 |
| Rate for the recovery | 106.56% | 82.48% | 95.88% | 85.58% | | 85.78% | 87.29% | | 88.78% |
| HQC(μg/mL) | 25 | 10 | 10 | 10 | | 10 | 10 | | 10 |
| The rate for the recovery | 114.68% | 88.94% | 101.51% | 89.94% | 100.11% | | 90.98% | 87.70% | |

**Table S4** Pearson correlation annlysis total SCFAs, acetic acid and propionic acid and time on the rotarod, mNSS tests, and the percentage of cerebral infarct volume

| **SCFAs** | **rotarod** | | **mNSS** | | **percentage of infarct volume** | |
| --- | --- | --- | --- | --- | --- | --- |
|  | **Pearson r** | **p-value** | **Pearson r** | **p-value** | **Pearson r** | **p-value** |
| Total SCFAs | 0.265 | 0.406 | -0.201 | 0.530 | -0.131 | 0.684 |
| acetic acid | 0.244 | 0.445 | -0.167 | 0.604 | -0.105 | 0.745 |
| propionic acid | 0.633 | 0.027 | -0.698 | 0.012 | -0.729 | 0.007 |

Significant correlations were determined based on < –0.5 Pearson r < 0.5 and *p* < 0.05.
