# Supplementary figures and images for: Effect of Electroacupuncture on Short-Chain Fatty Acids in Peripheral Blood after Middle Cerebral Artery Occlusion/Reperfusion in Rats Based on Gas Chromatography–Mass Spectrometry
Source: Mediators Inflamm. 2022 Aug 23;2022:3997947. doi: 10.1155/2022/3997947 (PMC9427317; doi:10.1155/2022/3997947)

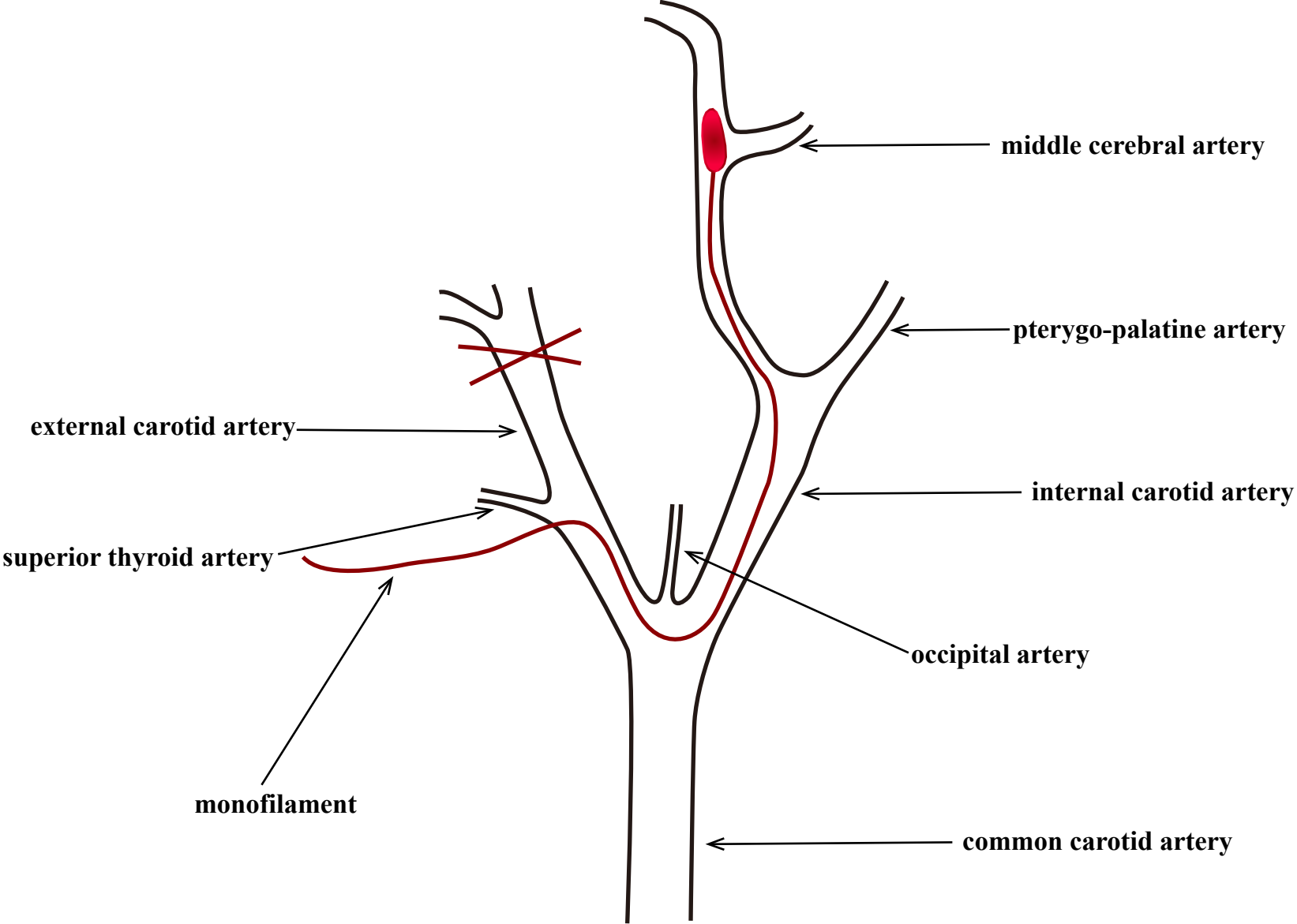

Supplement: Supplementary Materials — Figure S1. Schematic diagram of the carotid artery and monofilament insertion in rats. Figure S2 (a) Exposure of common, internal and external carotid arteries; (b) ligation of the distal common and external carotid arteries and a live knot in the proximal external and internal carotid arteries; (c) inserting the monofilament; and (d) fixed the monofilament. Figure S3. Overlapping chromatograms of QC samples. Figure S4. T2-weighted imaging signal changes before and after sham group. Table S1. Modified neurological severity score (mNSS). Table S2. Regression equation, linear range, precision, repeatability, limit quantitation, and stability for the determination of the SCFAs in peripheral blood (n = 6). Table S3. Rate for the recovery for the determination of standard sample (n = 6). Table S4. Pearson correlation analysis total SCFAs, acetic acid and propionic acid and time on the rotarod, mNSS tests, and the percentage of cerebral infarct volume. [file 3997947.f1.zip › FIGURE S1 (1).pdf]

A

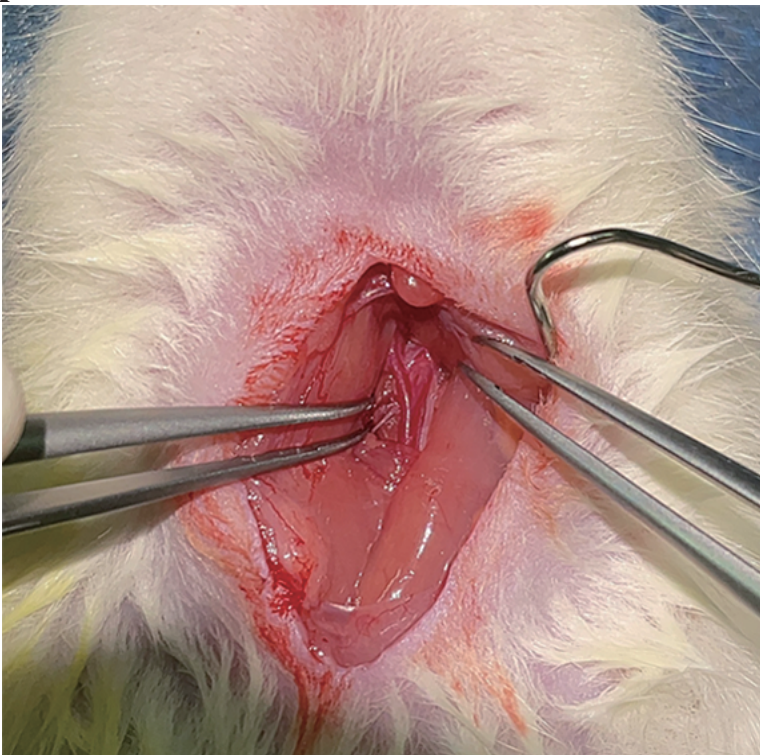

B

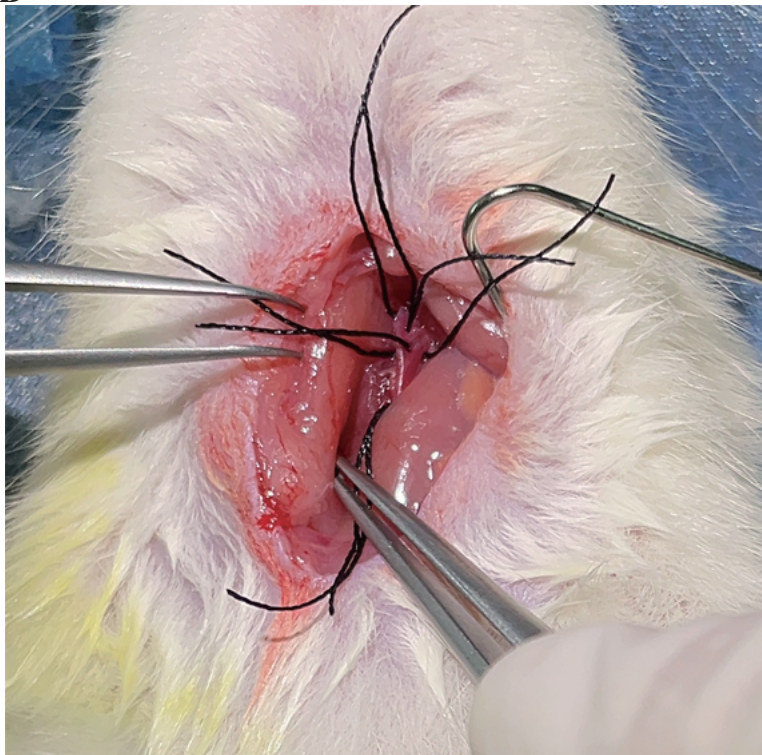

C

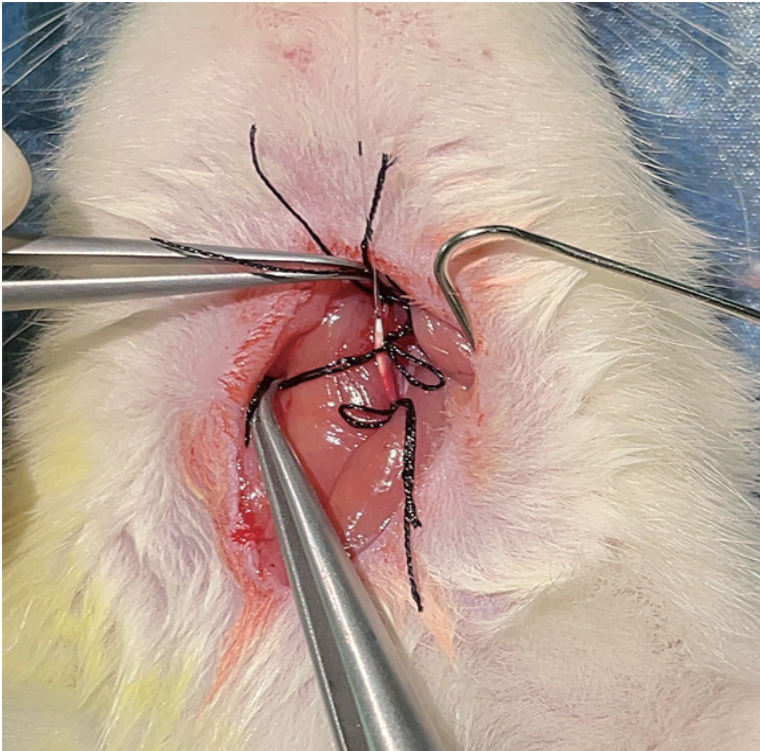

D

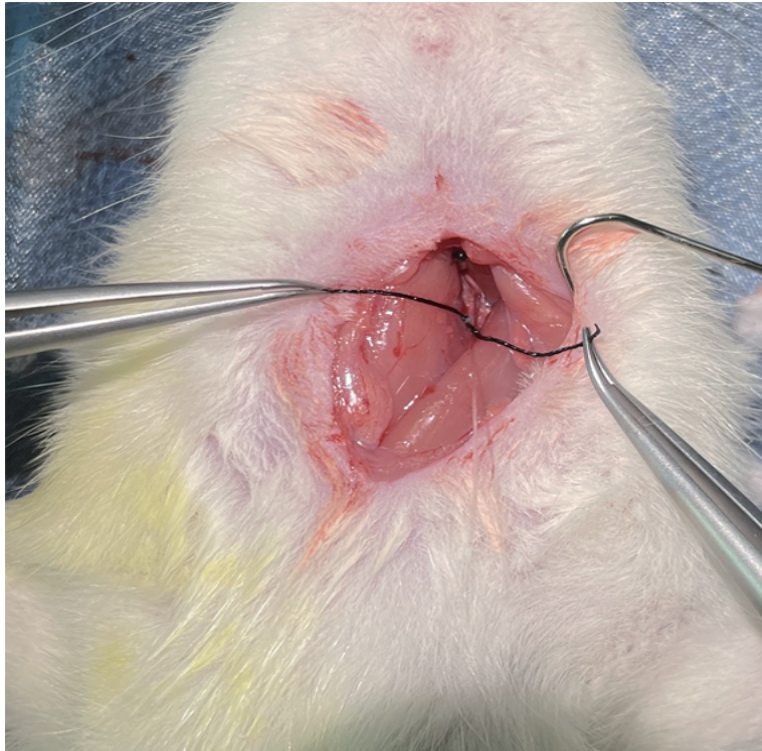

Supplement: Supplementary Materials — Figure S1. Schematic diagram of the carotid artery and monofilament insertion in rats. Figure S2 (a) Exposure of common, internal and external carotid arteries; (b) ligation of the distal common and external carotid arteries and a live knot in the proximal external and internal carotid arteries; (c) inserting the monofilament; and (d) fixed the monofilament. Figure S3. Overlapping chromatograms of QC samples. Figure S4. T2-weighted imaging signal changes before and after sham group. Table S1. Modified neurological severity score (mNSS). Table S2. Regression equation, linear range, precision, repeatability, limit quantitation, and stability for the determination of the SCFAs in peripheral blood (n = 6). Table S3. Rate for the recovery for the determination of standard sample (n = 6). Table S4. Pearson correlation analysis total SCFAs, acetic acid and propionic acid and time on the rotarod, mNSS tests, and the percentage of cerebral infarct volume. [file 3997947.f1.zip › FIGURE S2 (1).pdf]

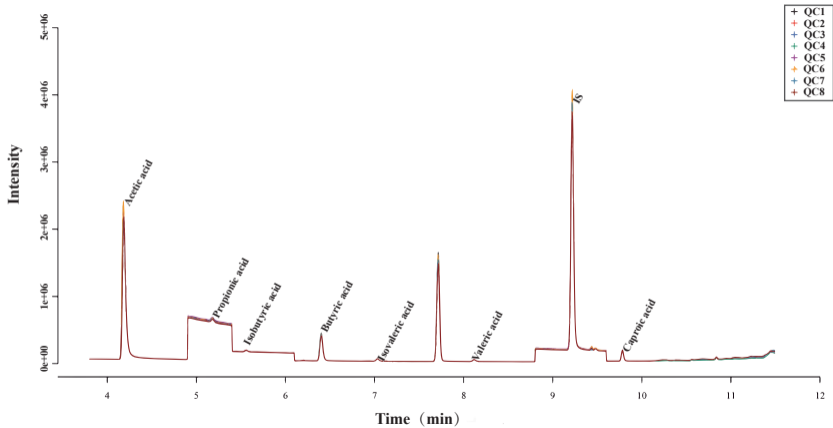

Supplement: Supplementary Materials — Figure S1. Schematic diagram of the carotid artery and monofilament insertion in rats. Figure S2 (a) Exposure of common, internal and external carotid arteries; (b) ligation of the distal common and external carotid arteries and a live knot in the proximal external and internal carotid arteries; (c) inserting the monofilament; and (d) fixed the monofilament. Figure S3. Overlapping chromatograms of QC samples. Figure S4. T2-weighted imaging signal changes before and after sham group. Table S1. Modified neurological severity score (mNSS). Table S2. Regression equation, linear range, precision, repeatability, limit quantitation, and stability for the determination of the SCFAs in peripheral blood (n = 6). Table S3. Rate for the recovery for the determination of standard sample (n = 6). Table S4. Pearson correlation analysis total SCFAs, acetic acid and propionic acid and time on the rotarod, mNSS tests, and the percentage of cerebral infarct volume. [file 3997947.f1.zip › FIGURE S3 (1).pdf]

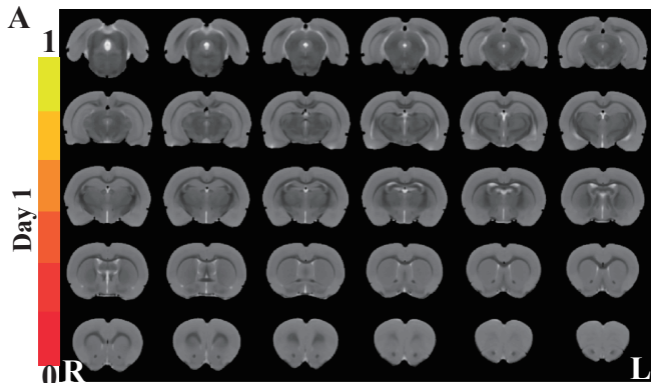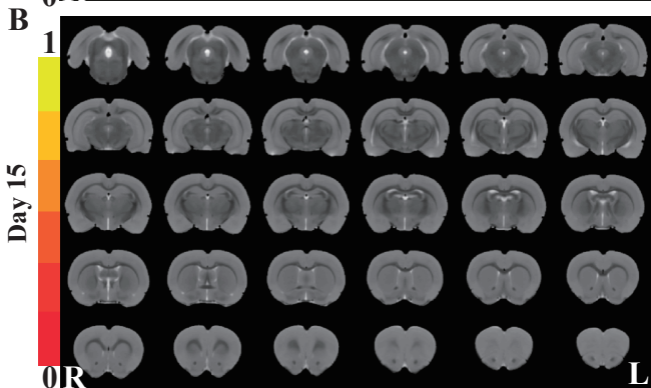

**Sham Group**

Supplement: Supplementary Materials — Figure S1. Schematic diagram of the carotid artery and monofilament insertion in rats. Figure S2 (a) Exposure of common, internal and external carotid arteries; (b) ligation of the distal common and external carotid arteries and a live knot in the proximal external and internal carotid arteries; (c) inserting the monofilament; and (d) fixed the monofilament. Figure S3. Overlapping chromatograms of QC samples. Figure S4. T2-weighted imaging signal changes before and after sham group. Table S1. Modified neurological severity score (mNSS). Table S2. Regression equation, linear range, precision, repeatability, limit quantitation, and stability for the determination of the SCFAs in peripheral blood (n = 6). Table S3. Rate for the recovery for the determination of standard sample (n = 6). Table S4. Pearson correlation analysis total SCFAs, acetic acid and propionic acid and time on the rotarod, mNSS tests, and the percentage of cerebral infarct volume. [file 3997947.f1.zip › FIGURE S4 (1).pdf]
